# Knowledge, attitudes and practices of grandmothers related to breastfeeding: a meta-synthesis

**DOI:** 10.1590/1518-8345.3097.3214

**Published:** 2020-02-14

**Authors:** Bárbara Helena de Brito Angelo, Cleide Maria Pontes, Gabriela Cunha Schechtman Sette, Luciana Pedrosa Leal

**Affiliations:** 1 Universidade Federal de Pernambuco, Recife, PE, Brasil.; 2 Universidade Federal de Pernambuco, Hospital das Clínicas, Núcleo Interno de Regulação, Recife, PE, Brasil.; 3 Universidade Federal de Pernambuco, Departamento de Enfermagem, Recife, PE, Brasil.

**Keywords:** Health Knowledge, Attitudes, Practice, Grandparents, Breast Feeding, Qualitative Research, Nursing, Health Education, Conhecimentos, Atitudes e Prática em Saúde, Avós, Aleitamento Materno, Pesquisa Qualitativa, Enfermagem, Educação em Saúde, Conocimientos, Actitudes y Práctica en Salud, Abuelos, Lactancia Materna, Investigación Cualitativa, Enfermeira, Educación em Salud

## Abstract

**Objective:**

Evaluate the knowledge, attitudes and practices of grandmothers that support or discourage the breastfeeding process.

**Method:**

This is a meta-synthesis based on the theoretical and methodological framework of meta-ethnography developed by Noblit and Hare. A critical evaluation of the articles was conducted using the consolidated criteria for reporting qualitative research (Coreq). Primary and secondary constructs derived from the results of nine articles were grouped into a new theory, leading to tertiary constructs that were presented in a diagram based on the functionality of Sanicola’s Social Network Theory.

**Results:**

Grandmothers know the benefits of breastfeeding, the importance of a special diet and proper hydration for the production of good quality milk, but report inadequacies in the treatment of breast complications and the need for breast milk complementation before the sixth month of life. These aspects were also observed: religious interference, opposition of ideas about breastfeeding, and family decisions based on the grandmother figure.

**Conclusion:**

Through knowledge, attitudes and practices, grandmothers, central figures in breastfeeding support, support their daughters and daughters-in-law in breastfeeding or discourage breastfeeding with contrary opinions and inadequate guidance.

## Abstract (pt)

**Objetivo:**

avaliar os conhecimentos, atitudes e práticas das avós relacionados ao suporte ou à contenção no processo de aleitamento materno.

**Método:**

metassíntese alicerçada no Referencial Teórico e Metodológico da Metaetnografia de Noblit e Hare. Na avaliação crítica dos artigos utilizou-se o *Consolidated criteria for reporting qualitative research* (Coreq). Os construtos primários e secundários, oriundos dos resultados dos nove artigos, foram agrupados em nova teoria, originando os construtos terciários que foram apresentados num diagrama construído com base na funcionalidade da Teoria de Rede de Sanicola.

**Resultados:**

as avós conhecem os benefícios da amamentação, a importância de uma dieta especial e hidratação adequada para produção de leite de boa qualidade, mas relatam inadequações no tratamento de intercorrências mamárias e necessidade de complementação do leite materno antes do sexto mês de vida. Houve interferência religiosa, oposição de ideias sobre amamentação e a centralidade das decisões familiares na figura das avós.

**Conclusão:**

as avós, figuras centrais no apoio à amamentação, através de conhecimentos, atitudes e práticas, oferecem suporte às suas filhas e noras para amamentarem ou promovem a contenção ao aleitamento materno com opiniões contrárias e orientações inadequadas.

## Abstract (es)

**Objetivo:**

evaluar los conocimientos, las actitudes y las prácticas de las abuelas en torno al soporte o la contención al proceso de lactancia materna.

**Método:**

metasíntesis basada en el marco teórico y metodológico de la metaetnografía de Noblit y Hare. En la evaluación crítica de los artículos, se utilizó el *Consolidated criteria for reporting qualitative research* (Coreq). Los constructos primarios y secundarios, provenientes de los resultados de nueve artículos, se agruparon en una nueva teoría, dando lugar a constructos terciarios que se presentaron en un diagrama elaborado con base en la funcionalidad de la Teoría de Red de Sanicola.

**Resultados:**

las abuelas conocen los beneficios de la lactancia, la importancia de una dieta especial e hidratación adecuada para la producción de leche de buena calidad, pero han presentado inadecuaciones acerca del tratamiento de las complicaciones mamarias y de la necesidad de complementación a la leche materna antes del sexto mes de vida. Hubo interferencia religiosa, contraste de ideas sobre lactancia materna y la centralidad de las decisiones familiares en la figura de las abuelas.

**Conclusión:**

las abuelas, figuras centrales en el apoyo a la lactancia materna por medio de los conocimientos, las actitudes y prácticas, brindan el soporte a la lactancia de sus hijas y nueras en torno a la lactancia o promueven la contención de la lactancia materna con opiniones contrarias y orientaciones inadecuadas.

## Introduction

Breastfeeding is intrinsic to humans; however, it is directly influenced by political, economic, social and cultural aspects, which are passed down to generations. This fact may determine whether the social network will support or discourage breastfeeding, affecting the mother’s choices regarding her newborn’s feeding^(^ 1 ^-^ 2 ^)^.

Among family members, grandmothers are significant figures for transferring information and experiences who influence the mother’s breastfeeding decision. In addition, in the postpartum period, grandmothers often spend more time with their daughters and daughters-in-law, providing emotional and financial support, taking care of the mother, the baby and older children, and helping with household chores^(^ 3 ^)^.

Evidence of grandmother participation in the construction of the breastfeeding process shows that having grandmothers as supporters in maintaining this practice is not always an easy task^(^ 3 ^-^ 4 ^)^. According to health professionals, the concepts grandmothers have about breastfeeding and human milk are more difficult to change than those of their own daughters^(^ 4 ^)^.

Grandmothers have knowledge that has been validated by their experiences, becoming socially accepted, valued and respected, and leading to attitudes and practices that can support or discourage breastfeeding^(^ 5 ^)^. In this context, such knowledge, attitudes and practices (KAPs) of grandmothers should be analyzed in their different dimensions affecting the process of breastfeeding.

Knowledge can be defined as the understanding of a subject, which results from experience or learning, applied when solving problems or developing concepts. Attitude is linked with the affective domain, whose judgmental tendency is based on feelings, predispositions and beliefs about a given topic. Practice refers to how an action is performed based on one’s knowledge and attitudes^(^ 6 ^-^ 7 ^)^.

The analysis of KAPs of grandmothers related to breastfeeding may place the scientific knowledge of nurses closer to the popular knowledge, supporting the development of actions to fulfill the needs of women and their social support networks. Data from this meta-synthesis may guide clinical practices and health education activities to minimize breastfeeding myths and beliefs. Then, this study aimed to evaluate the knowledge, attitudes and practices of grandmothers that support or discourage breastfeeding.

## Method

This review is a meta-synthesis, a study method that thoroughly analyzes the theory, methods and results obtained in qualitative studies^(^ 8 ^)^. This meta-synthesis was built using the theoretical and methodological framework of meta-ethnography developed by Noblit and Hare, which has seven steps: 1) define the area of interest, formulate a study question and develop search strategies; 2) define relevant studies for the study objective; 3) read the studies, recording important information; 4) determine how the studies are related; 5) compare the studies with each other; 6) synthesize the findings; and 7) express the synthesis by presenting the results^(^ 9 ^)^.

The study aims to answer the following question: “What are the knowledge, attitudes and practices of grandmothers that support and/or discourage the breastfeeding process?” Database search was conducted using Health Sciences Descriptors (DeCS) and their correspondents in the Medical Subject Headings (MeSH) in Portuguese, English and Spanish. The following descriptors were defined: “breastfeeding,” “grandmothers,” “qualitative research,” “knowledge, attitudes and practice (KAPs) in health”.

The bibliographic research was conducted in Medline, Scopus, Cuiden, Lilacs, Bdenf databases and SciELO virtual library; three from the health care area, two from multidisciplinary areas, and one from nursing to ensure a broad national and international search.

Search using “KAP (AND) breastfeeding,” “KAP (AND) grandmothers,” “breastfeeding (AND) grandmothers,” “breastfeeding (AND) qualitative research,” “grandmothers (AND) qualitative research,” “breastfeeding (AND) grandmothers (AND) qualitative research,” “breastfeeding (AND) grandmothers (AND) KAP,” and “breastfeeding (AND) grandmothers (AND) KAP (AND) qualitative research,” identified 3,693 articles, which were screened according to the inclusion criteria.

Original articles were included when fulfilling the following criteria: they should be exclusively qualitative studies in Portuguese, English and Spanish investigating knowledge, attitudes and practices of grandmothers related to support or discourage to breastfeeding, and whose participants were mothers and/or grandmothers. No time limit was defined, as it sought to investigate how the KAPs of grandmothers behaved over the years.

Editorials, letters to the editor, reflective and review studies, end-of-course assignment, theses and dissertations were excluded. Duplicate articles were considered only once, respecting the order of search in databases and virtual library.

An instrument adapted from the Joanna Briggs Institute was used for data extraction, which includes title, authors, country, year, place of publication, phenomenon of interest, study objectives, population, methodology, theory (name and description), results, conclusions, and comments of reviewers. Data collection site was added to the instrument^(^ 10 ^)^.

The articles were critically analyzed using the Consolidated criteria for reporting qualitative research (Coreq). Coreq is a checklist containing 32 items grouped into three domains: research team and reflexivity; study design; analysis and results^(^ 11 ^)^. To avoid the exclusion of relevant studies, after Coreq application, a careful evaluation was conducted by two researchers with knowledge about the theme and qualitative research, first individually, and then a subsequent meeting was held for consensus between them. Items that were not resolved by consensus at the first meeting were discussed at a second meeting with two other researchers. No article was removed in this step.

An interpretative synthesis of the results, based on the theoretical and methodological framework of meta-ethnography developed by Noblit and Hare^(^ 9 ^)^, was developed through reciprocal translation of the chronologically organized articles, so that the key concepts identified by thematic analysis of the first article were confronted with the concepts of the subsequent article, feeding the interpretation, and then successively until all articles were analyzed. This step was performed by two independent reviewers and, in the absence of a consensus, two additional reviewers analyzed the item causing disagreement.

The key concepts were allocated to two columns; the first column had concepts from the study participants’ understanding (first-order constructs) and the second had concepts from the study authors’ interpretation of participants’ understanding (second-order constructs). A synthesis and analysis of primary and secondary constructs originated the third-order or tertiary constructs, which were organized into a new theory resulting from the meta-synthesis^(^ 12 ^)^.

When analyzing the tertiary constructs, the knowledge, attitudes and practices of grandmothers were favorable or unfavorable to successful breastfeeding. Sanicola’s Social Network Theory^(^ 13 ^)^ was used to guide such dichotomy and the construction of thematic categories, as it postulates the role of the members of this network can be supportive or discouraging. Therefore, articulated with the tertiary constructs (knowledge, attitudes and practices), the thematic categories express the type of support or discourage from grandmothers to breastfeeding.

For contextualization purposes, the first-order constructs were represented by the statements extracted from the meta-synthesis studies, identifying the authorship - mother or grandmother - and the number corresponding to the article reference. The second-order constructs were presented by concepts extracted directly from the studies and identified by the bibliographic reference. The tertiary constructs are indicated in italics.

The development of this meta-synthesis was guided by Entreq (Enhancing Transparency in Reporting the Synthesis of Qualitative Research), a checklist containing 21 items grouped into five main domains: introduction, methods, literature search and selection, appraisal and synthesis of findings^(^ 14 ^)^.

## Results

After searching with the descriptors, 3,693 articles were found; of these, 919 were removed as they were duplicate articles, leading to 2,774 remaining articles. After applying the inclusion and exclusion criteria and reading the titles, 46 articles were selected for abstract reading; and only then were selected for full-text reading. However, of these, one article had no full text, despite attempts to acquire it through the Central Library of the Universidade Federal de Pernambuco and contact with the corresponding author.

**Figure 1:**
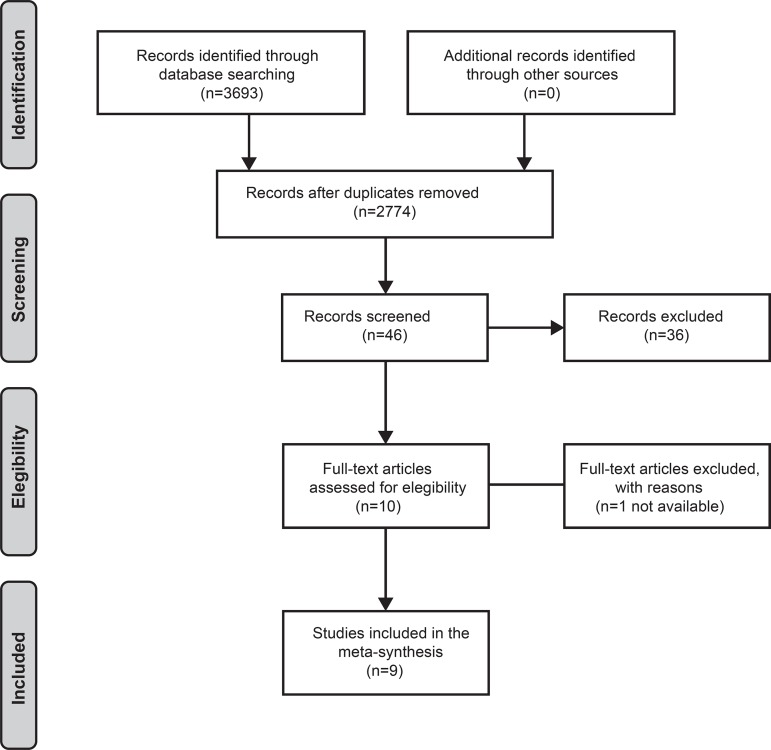
PRISMA flow diagram for the selection of articles comprising the study sample^(^ 15 ^)^

The reference lists of the articles were analyzed to identify other publications that could answer the study question, but no other study was selected at this stage. Therefore, nine articles were submitted to a critical evaluation. Figure 1 details the sample selection process.

The studies were conducted in the United Kingdom^(^ 16 ^)^, Nepal^(^ 17 ^)^, Malawi^(^ 18 ^)^, United States^(^ 19 ^)^, Australia^(^ 20 ^)^, Brazil^(^ 21 ^-^ 22 ^)^, Pakistan^(^ 23 ^)^ and Myanmar^(^ 24 ^)^. Regarding the study participants, four studies interviewed grandmothers only^(^ 16 ^-^ 17 ^,^ 20 ^,^ 22 ^)^ and two interviewed mothers only^(^ 19 ^,^ 21 ^)^, the other studies included grandmothers and mothers^(^ 18 ^)^, mothers and fathers^(^ 23 ^)^, and mothers, fathers and grandmothers^(^ 24 ^)^. Data were mostly collected at the homes of participants^(^ 16 ^-^ 17 ^,^ 21 ^,^ 23 ^-^ 24 ^)^, and some at community centers^(^ 16 ^,^ 18 ^,^ 23 ^)^, clinics and hospitals^(^ 19 ^,^ 22 ^)^.

The most common data collection technique was a combination of focus group and semi-structured interview^(^ 16 ^-^ 18 ^,^ 20 ^,^ 23 ^)^, followed by semi-structured interview alone^(^ 21 ^,^ 24 ^)^, focus group^(^ 19 ^)^, and questionnaire^(^ 22 ^)^. Data were processed using content analysis^(^ 17 ^,^ 19 ^,^ 22 ^,^ 24 ^)^, thematic analysis^(^ 20 ^-^ 21 ^,^ 23 ^)^ and Grounded Theory^(^ 18 ^)^. One article had no technique description^(^ 16 ^)^. Regarding the theoretical and methodological framework, the Phenomenological Method^(^ 17 ^,^ 23 ^)^ and the Theory of Culture Care Diversity and Universality^(^ 21 ^-^ 22 ^)^ were the most frequent ones, followed by the Grounded Theory^(^ 18 ^)^ and the Conceptual Model^(^ 24 ^)^. Three articles did not describe the theory that guided the study development^(^ 16 ^,^ 19 ^-^ 20 ^)^. Figure 2 shows the characteristics of the studies.

**Figure 2:** Characteristics of the primary studies included in the meta-synthesis. Recife, PE, Brazil, 2016

Authors, year of publication and country	Theoretical and methodological framework	Data collection site	Data collection technique	Study participants	Type of analysis
Ingram J, Johnson D, Hamid N 2003^(^ 16 ^)^ United Kingdom	Not described	Health center and homes	Focus group and interviews	14 grandmothers	Not described
Masvie H 2005^(^ 17 ^)^ Nepal	Phenomenological Method	Homes and outdoor area in the village	Focus group and semi-structured interview	31 grandmothers	Content analysis according to Miles and Huberman, 1994
Kerr RB , Dakishoni L, Shumba L et al., 2008^(^ 18 ^)^ Malawi	Grounded Theory	Community	Focus group and semi-structured interview	4 grandmothers and 8 mothers	Grounded Theory based on Ryan; Bernard, 2009
Grassley J, Eschiti V 2008^(^ 19 ^)^ United States	Not described	Clinic and hospital	Focus group	30 mothers	Content analysis according to Mayan, 2001
Reid J, Schmied V, Beale B 2010^(^ 20 ^)^ Australia	Not described	Not described	Focus group and semi-structured interview	11 grandmothers	Thematic analysis. No author mentioned as reference.
Gross FM, Van der Sand ICP, Girardon-Perlini NMO et al. 2011^(^ 21 ^)^ Brazil	Theory of Culture Care Diversity and Universality	Homes	Semi-structured interview	11 mothers	Thematic analysis based on Mynayo, 2007
Silva LR, Cruz LA, Macedo EC et al. 2013^(^ 22 ^)^ Brazil	Theory of Culture Care Diversity and Universality	Rooming-in care	Questionnaire	20 grandmothers	Content analysis. No author mentioned as reference.
Premji S, Khowaja S, Meherali S et al. 2014^(^ 23 ^)^ Pakistan	Phenomenological Method	Homes and community centers	Semi-structured interview and focus group	10 mothers and 8 fathers	Thematic analysis based on Colaizzi, 1978
Thet MM, Khaing EE, Diamond-Smith N et al. 2016^(^ 24 ^)^ Myanmar	Conceptual Model	Homes	Semi-structured interview	24 mothers, 10 fathers and 10 grandmothers	Content analysis according to Miles and Huberman, 1994

**Figure 3:**
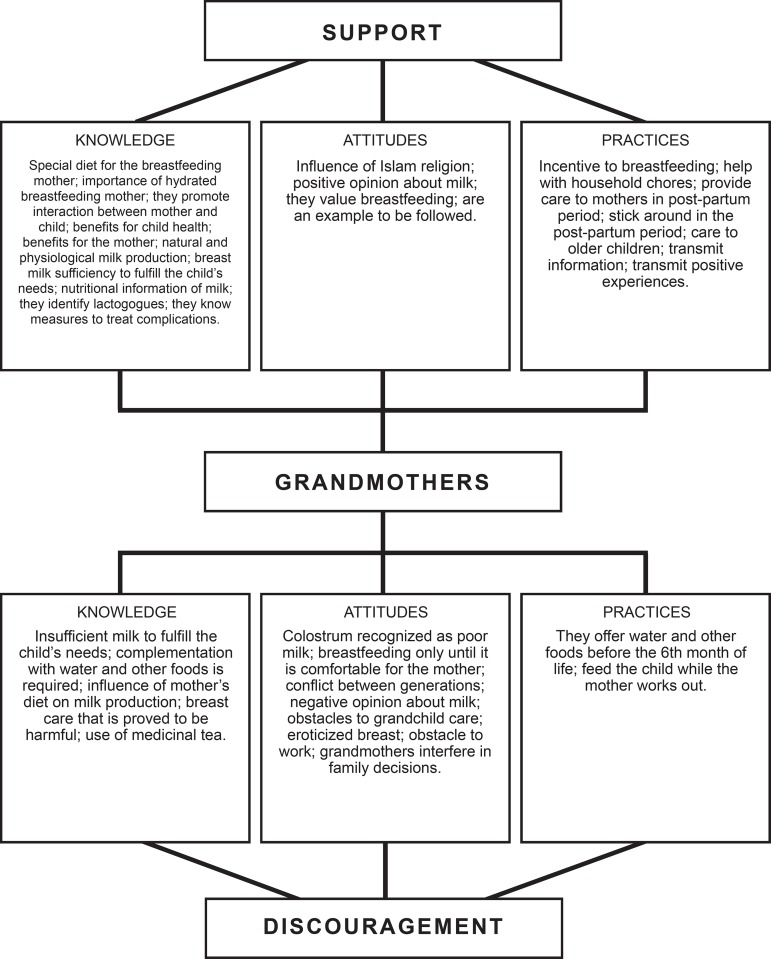
Support and discouragement of the social network articulated with the knowledge, attitudes and practices of grandmothers in breastfeeding. Recife, PE, Brazil, 2016

From the synthesis and analysis of primary and secondary constructs, a Theory was built that shows the type of support offered by grandmothers in the breastfeeding process. Grandmothers are in the central subjects of the diagram, who can support or discourage breastfeeding through their knowledge, attitudes and practices. Figure 3 illustrates the Theory. 

The thematic categories linked with the tertiary constructs (knowledge, attitudes and practices) will be represented by the support and/or discouragement grandmothers provide in the context of breastfeeding.

### Knowledge of grandmothers

#### Influence of special diet and proper hydration on milk quality

Grandmothers identified a specific diet is required, including green vegetables, chicken, meat and nuts, as well as hydration for the maintenance of the mother’s well-being and production of good quality milk^(^ 16 ^-^ 17 ^,^ 21 ^-^ 22 ^)^. They often attributed special value to some products, lactogogues, which could increase the quality and quantity of breast milk, such as *canjica*, dark beer and mate^(^ 22 ^)^. Also regarding the mother’s diet and milk quality, grandmothers stated nursing mothers should be cautious about foods that can cause baby colic^(^ 19 ^)^.

*For the mother the following foods are prepared: rice porridge, chicken with condiments, butter, milk, spicy rice, oil and ghee (butter). (GRANDMOTHER)(* ^(^ 17 ^)^

*Our food has special ingredients... they say if mothers eat this type of food, their milk will be nutritious. If not, where does the milk come from? (GRANDMOTHER)(* ^(^ 17 ^)^

#### Benefits of breast milk

Breast milk was mentioned as a natural and nutritious food^(^ 17 ^)^, whose production is physiological and sufficient to fulfill the child’s needs, making them healthier with reduced allergies, eczema, stomach problems, greater intelligence and stronger bones^(^ 16 ^,^ 22 ^,^ 24 ^)^. Grandmothers also recognized the immunological properties of milk^(^ 21 ^)^. Regarding the benefits for the mother, breastfeeding was associated with lower risk of breast cancer, pregnancy prevention and weight loss, also representing a connection between mother and child^(^ 16 ^)^.

*Even if the milk doesn’t come out, the baby remains with the mouth to the breast, and later the milk will come out. No other food is required. (GRANDMOTHER)(* ^(^ 17 ^)^

*The child’s grandmother told me that the longer I can breastfeed the child with breast milk only, until six months, I shouldn’t give anything else, it’s healthier for the child. That the breast milk is like a medicine, it doesn’t cause disease, nothing, it gives more immunity. So she encouraged me to breastfeed him. (MOTHER)(* ^(^ 21 ^)^

*... it’s an act of love. (GRANDMOTHER)(* ^(^ 22 ^)^

#### Treating complications (in)adequately

For the treatment of breast engorgement and healing of sore nipples, grandmothers mentioned milk extraction^(^ 16 ^)^. In other studies, grandmothers emphasized scientifically proven care that are harmful to breasts, such as cleaning at each feeding session, wiping the breasts with alcohol, using ointments to treat cracks, warm compress to treat engorgement, and massage to prevent nipple obstruction^(^ 17 ^,^ 22 ^,^ 24 ^)^.

*My husband took the syringe and went to his mother’s home, as she has more experience, and he learned how to cut the syringe and pull the milk; then he came back and made it on me. (MOTHER)(* ^(^ 24 ^)^

[...] *I used to apply alcohol to clean the breasts* [...] *. I used to apply warm compresses for breast engorgement. (GRANDMOTHER)(* ^(^ 22 ^)^

#### Complementary feeding required

According to the grandmothers, children need water before the sixth month of life to quench thirst, moisturize the skin, extend the time between breastfeeding sessions and, when mixed with sugar, treat asthma^(^ 16 ^,^ 19 ^)^. Complementation with porridge and other foods, in their opinion, should be offered to children who are born crying and hungry, if the mother has little milk or needs to rest and while milk does not come out^(^ 16 ^,^ 18 ^,^ 19 ^,^ 21 ^,^ 23 ^)^. Teas were frequently used as home remedy to calm a restless child^(^ 21 ^)^.

[If] *a child is born and crying, then we say the child is born hungry, then we give dawale (herb mix) which is water... Then if the baby keeps crying, we also give porridge. (GRANDMOTHER)(* ^(^ 18 ^)^

*Oh, no one can really breastfeed. The milk just doesn’t work anymore. I know you are trying, but, you know, you will have no milk. (MOTHER)(* ^(^ 19 ^)^

*Oh, something, tea is good, it helps the child calm down. (MOTHER)(* ^(^ 21 ^)^

### Attitudes of grandmothers

#### Religious influence

Islam has a positive influence on breastfeeding as it states milk is Allah’s gift to a woman, and if she refuses to offer it to the child, she will have to respond to it. For this reason, Muslim grandmothers encourage breastfeeding for two years or more, unlike Hindus who had no religious references about the time to maintain or discontinue this practice^(^ 16 ^)^.

#### Opposed ideas about breastfeeding

Appreciation, positive opinion and approval were also supportive attitudes in the mother’s decision to breastfeed^(^ 19 ^-^ 22 ^)^. The statements of nursing mothers showed the importance given by them to supporting words, examples to be followed or encouragement from their grandmothers^(^ 19 ^)^.

*My mother used to say: ‘this is the best thing you can do, she really enjoyed breastfeeding her children. (MOTHER)(* ^(^ 19 ^)^

*I used to feed my dolls and she* [my mother] *encouraged me to do it [she said]: ‘one day you will grow up, you will be a mother, and you will breastfeed your children (MOTHER)(* ^(^ 19 ^)^

On the other hand, some grandmothers had a negative opinion about breastfeeding by associating colostrum with bad food. Among the grandmothers of different ethnicities represented in the articles, the Nepalese women have greater confidence in breast milk; however, they distinguish the first milk, *khil*, described as the dirt that clogged the breast, from colostrum or *bigouti*, which comes later. For grandmothers born in other Asian countries, such as Pakistan, Bangladesh and India, colostrum is identified as old milk stored in the breast for a long time and that should be discarded^(^ 17 ^)^.

*First, the breast is washed. Khil is washed and thrown away and then we feed the baby. (GRANDMOTHER)(* ^(^ 17 ^)^

*Colostrum is old milk that has been stored in the breast for a long time. (GRANDMOTHER)(* ^(^ 16 ^)^

Another cultural judgment is eroticized breast, leading to shame or disapproval of breastfeeding in public. By associating breasts with pleasure, Malawi’s grandmothers believe that couples should refrain from sexual activities in the first year after a child is born, during breastfeeding^(^ 18 ^)^.

*My mother told me breastfeeding was disgusting... she was it was unpleasant, inadequate. (MOTHER)(* ^(^ 19 ^)^

*It’s because young women don’t respect old women, they’re busy giving in to their husbands. This is the cause of the problem. (GRANDMOTHER)(* ^(^ 18 ^)^

#### Grandmothers as the central figure of family decisions

In different cultures, given the social role of the matriarch, the inherent aspects of child feeding are subjected to the opinion of grandmothers, generating conflicts when their concepts are divergent^(^ 18 ^,^ 20 ^,^ 23 ^)^. One study highlighted the opinion of the paternal grandmothers about the most adequate moment to stop breastfeeding and introduce other foods, which was observed by the mothers as they feared their children would be taken away from them^(^ 18 ^)^.

*I have a mother in law... I ask her what I should do, if she forbids me to do something, I don’t do it. (MOTHER)(* ^(^ 23 ^)^

Believing that they are doing a good thing for their daughters and daughters-in-law, grandmothers recommend breastfeeding should be maintained as long as it is comfortable for mothers and then complemented feeding should be started, so that they can rest or work out, preventing the trauma of a transition between breastfeeding and bottle feeding. In addition, breastfeeding was seen as an obstacle to grandchild care and the creation of a bond between them.

*My mother and mother-in-law said: ‘It’s not good for you to breastfeed every three hours. You can give the baby some formula. Let me do it so you can sleep.’ (MOTHER)(* ^(^ 19 ^)^

*... One disadvantage the mother-in-law saw was that she couldn’t stay with the baby when I wasn’t there. (MOTHER)(* ^(^ 19 ^)^

### Practices of grandmothers

#### Transmitting information

Grandmothers are responsible for passing on information and experiences in the family, being identified as the first source of advice to be sought. Grandmothers provided information to their daughters and daughters-in-law about proper position of the child next to the breast, the need to breastfeed according to the demand, exposure of the breast to sunlight, start of breastfeeding in the first hour of life, and hand cleaning before breastfeeding^(^ 16 ^-^ 22 ^)^.

*If I have a doubt, the first thing I do is to ask her [grandmother of the child] if it is good or not. (MOTHER)(* ^(^ 21 ^)^

*I feel safe, I feel safer, because she has experience... (MOTHER)(* ^(^ 21 ^)^

#### Two sides of support

In the post-partum period, grandmothers stayed longer with the nursing mother^(^ 21 ^)^ helping with household chores, providing care to mothers, newborns and older children^(^ 18 ^,^ 24 ^)^. With the support in the post-partum period and care provided by grandmothers while mothers work out, they give water and other foods to children before the sixth month of life, even though health professionals recommend exclusive breastfeeding.

*My mother helped me a lot when I had my children, I want to do the same thing for my children. (MOTHER)(* ^(^ 20 ^)^

*You can hear at the hospital, but when the child cries, the mother-in-law will ask her to prepare and feed the child with porridge. (MOTHER)(* ^(^ 18 ^)^

## Discussion

The Theory resulting from this meta-synthesis states that the knowledge, attitudes, and practices of grandmothers guide the breastfeeding process of their daughters and daughters-in-law, provide the necessary support for successful breastfeeding and/or promote discouragement seen in early discontinuation. This Theory was based on studies published in five different continents, with similarities between them and intracontinental particularities due to the existence of distinct ethnic, cultural and religious groups, with emphasis on the type of food that should be eaten by the mother. 

The greatest similarity found in the results was an association of a special diet for mothers with milk composition and quality^(^ 16 ^-^ 17 ^,^ 21 ^-^ 22 ^,^ 24 ^)^. In this context, knowledge promoted support through broad recommendations of grandmothers regarding the foods, beverages, and special products that nursing mothers should eat to produce milk of higher quality and in larger amounts. The foods offered to nursing mothers varied according to local culture, but were unanimous in terms of providing mothers with the best food available.

Although the dietary habits of mothers have little or no effect on most nutrients in human milk^(^ 25 ^)^, the literature recognizes the existence of artificial or natural substances that support milk production^(^ 26 ^-^ 27 ^)^. However, many of the foods mentioned by grandmothers as lactogogues, such as *canjica*, black beer and mate^(^ 21 ^-^ 22 ^)^, have no scientific explanation, but due to the importance of grandmothers, they may ensure mother’s self-efficacy and willingness to breastfeed, consequently increasing milk production.

Another aspect in the knowledge of grandmothers that supported breastfeeding was the idea that breast milk is a nutritious food that gives the child strength, health and immunity^(^ 21 ^-^ 22 ^,^ 24 ^)^. Regarding the benefits, breastfeeding was related to higher level of intelligence, in agreement with data from a Brazilian study started in 1982 that observed, after 30 years, better performance in intelligence tests in adults who had been breastfed^(^ 28 ^)^. In addition, breastfeeding was recognized as an act of love^(^ 22 ^)^ that promotes an affective bond between the mother and the child, enabling intimacy, exchange of affection and feelings of safety and protection^(^ 29 ^)^.

In most articles, although grandmothers were positive about breastfeeding for the mother and child^(^ 16 ^,^ 22 ^,^ 24 ^)^, they recommend the introduction of water and other foods before the sixth month of life^(^ 19 ^,^ 21 ^)^, as they are unaware that such practices negatively affect the time of exclusive breastfeeding and/or total breastfeeding. Grandmothers believe medicinal tea should be used to calm down or treat any discomfort of the child, such as dehydration and colic^(^ 21 ^-^ 22 ^)^.

Only one study reported grandmothers supporting breast milk as a natural, sufficient, necessary and irreplaceable food to fulfill the child’s nutritional demands. For these grandmothers in Nepal, breastfeeding should start shortly after birth and no other foods should be given to the child in the first months^(^ 17 ^)^.

The opinion of grandmothers about the right moment to give water and other foods to their grandchildren may support and/or discourage breastfeeding, as their knowledge is validated in their own experiences and culturally accepted, influencing decisions about child nutrition^(^ 30 ^)^.

The opinion of grandmothers about the quality and quantity of breast milk is a crucial point in supporting or discouraging the breastfeeding process. They instinctively tend to fulfill the child’s needs so, as long as crying is associated with the idea of hunger due to weak and insufficient milk, they will tend to offer some food which, in their minds, was able to feed other family members who are today strong and healthy, no matter if it is artificial milk, porridge or another food.

Knowledge that discourages breastfeeding also includes inadequate treatment of breast complications when grandmothers mentioned the use of alcohol and warm water^(^ 22 ^)^, which may harm the skin. Breast problems such as trauma and engorgement are common causes of early discontinuation and, if not properly treated, can be the source of infections and systemic problems requiring hospitalization^(^ 31 ^)^.

Passing on health information that is not scientifically proven or in disuse is a frequent situation between generations and harmful to a child’s health. For health professionals, beliefs, myths and misconceptions about breastfeeding are the result of lack of knowledge about this theme^(^ 32 ^)^.

Some knowledge that is harmful to breastfeeding is closely related to judgments based on beliefs, such as the representation of breasts as inappropriate and private organs. In contrast, for Muslims, breast milk is a divine gift and should be offered to the child; otherwise mothers will be responsible for their actions before Allah. In Brazil, it is common sense that the puerperal breast is the primary source of food to the detriment of a sexual organ notion^(^ 33 ^)^.

Attitudes about breastfeeding are influenced by the historical, social and cultural context of each family, showing a mother’s decision to breastfeed has an influence of tradition, orientation and encouragement^(^ 34 ^)^. Then, maintaining a positive opinion of grandmothers about breastfeeding through appreciation and example in stories or entertaining activities encourage mothers to start or maintain exclusive breastfeeding until the sixth month of life and continuously over the first two years of the child’s life.

On the other hand, some grandmothers believe colostrum is old milk that has been stored for a long time in the breast and, therefore, should be discarded, showing poor knowledge about milk composition and relevance^(^ 16 ^-^ 17 ^)^. Compared to mature milk, colostrum has a higher amount of protein and less energy, fat and lactose, which are essential for early life^(^ 35 ^)^. In addition, stimulating a child to maintain skin contact with the mother and breastfeeding in the first hour of life are protective factors for the maintenance of breastfeeding^(^ 36 ^)^.

Still regarding the attitude of breastfeeding discouragement, for grandmothers who take care of the child while their daughters and daughters-in-law work out, complemented feeding was seen as a tool to avoid the trauma of interrupting exclusive breastfeeding at the moment mothers would return to work. Understanding that going back to work is a risk factor for interrupting exclusive breastfeeding^(^ 37 ^)^, instructing mothers and grandmothers about breast milk extraction, storing and offering is a safe and inexpensive option.

Opposed ideas about breastfeeding indicate that it is not enough for nursing mothers to have knowledge. In some places, mothers have little autonomy and the opinion of grandmothers influences family decisions^(^ 18 ^)^, showing that grandmother education can be as relevant as mother education, which reinforces the importance of family history^(^ 38 ^-^ 39 ^)^.

The central role assumed by grandmothers in the Theory is due to the recognition of their influence on breastfeeding, which will be transmitted through supportive practices. Support is a comprehensive verb, related to counseling, advising, providing information, demonstration, examples, sharing of stories and beliefs^(^ 34 ^)^.

Family support plays an important role in a woman’s decision to breastfeed. Women whose families supported exclusive breastfeeding are 8.21 times more likely to start and continue breastfeeding^(^ 40 ^)^. In a study with more than 2,000 women conducted in the United States to investigate an association between the opinion of family members and health professionals in the success of breastfeeding four weeks after birth, the mothers who believed family members or health professionals preferred breastfeeding were more likely to start breastfeeding^(^ 41 ^)^.

However, support is provided in a hybrid manner. While grandmothers transmitted information and performed actions that allowed nursing mothers to have more time for themselves and the newborn^(^ 16 ^)^ by assuming household chores and care for the newborn and older children^(^ 18 ^,^ 24 ^)^, they, on the other hand, acted as an obstacle to breastfeeding by recommending water, teas and other foods to the child. For this reason, encouraging breastfeeding in the family context should be a priority among nurses and other health professionals. Recognizing the family habits in the puerperal period helps health professionals plan educational practices consistent with the local culture.

Educational activities are more likely to change paradigms with specific messages for the targeted context, respecting supporters of the belief system: the older members^(^ 32 ^)^. If grandmothers are well informed about the aspects related to breastfeeding, their knowledge, attitudes and practices will support nursing mothers and directly result in increased breastfeeding rates and a better quality of this experience that involves the mother, child and family. 

Despite the systematization used in the search and selection of articles included in this meta-synthesis, it is impossible to cover all studies published about this theme. Another study limitation was the lack of access to full transcription of participant speeches of the primary articles, which could provide a deeper analysis of the studied effect. In addition, the lack of information between the breastfeeding experience and the data collection period may allow recall bias.

## Conclusion

In the new Theory produced in this study, maternal and paternal grandmothers are the central figure of the nursing mother’s social network, performing supportive and/or discouraging roles in the breastfeeding process, which are expressed in their knowledge, attitudes and practices.

The Theory shows that breastfeeding transcends the practice of feeding a child, since it is inserted in macro contexts: historical, political, media, cultural and social circumstances, passed on between generations.

Health professionals, especially nurses, should position themselves emphatically and respectfully in clinical practice, planning and performing educational actions. Family particularities of those involved should be valued to contribute to proper knowledge, attitudes and practices for the start and maintenance of breastfeeding, consequently promoting increases in exclusive and total breastfeeding rates.

Regarding the grandmother relationship with the various actors who may be involved in the breastfeeding process, the results of this meta-synthesis refer only to mothers, and no influence of grandmothers was identified on the knowledge, attitudes and practices of the child’s father. Similar studies are recommended to investigate the participation of other significant members in the breastfeeding mother’s social network.

## References

[1] Caminha MFC, Cruz RSBL, Acioly VMC, Nascimento RR, Azevedo PTACC, Cabral de Lira PIC (2015). Risk factors for not breastfeeding: a case - control
study. Rev Bras Saúde Matern Infantil.

[2] Wilhelm LA, Demori CC, Alves CN, Barreto CN, Cremonese L, Ressel LB (2015). The experience of breastfeeding in women's perspective:
contributions to nursing. Rev Enferm UFSM.

[3] Angelo BHB, Pontes CM, Leal LP, Gomes MS, Silva TA, Vasconcelos MGL (2015). Breastfeeding support provided by grandmothers: an integrative
review. Rev Bras Saúde Matern Infantil.

[4] Bernie K (2014). The Factors Influencing Young Mothers' Infant Feeding Decisions:
The Views of Healthcare Professionals and Voluntary Workers on the Role of
the Baby's Maternal Grandmother. Breastfeeding Med.

[5] Prates LA, Schmalfuss JM, Lipinski JM (2015). Social support network of post-partum mothers in the practice of
breastfeeding. Esc Anna Nery.

[6] Nicolau AIO, Ribeiro SG, Lessa PRA, Monte AS, Bernardo EBR, Pinheiro AKB (2012). Knowledge, attitude and practices regarding condom use among
women prisoners: the prevention of std/hiv in the prison
setting. Rev Esc Enferm USP.

[7] Marinho LAB, Costa-Gurgela MS, Cecattia JG, Osisb MJD (2003). Knowledge, attitude and practice of breast self-examination in
health centers. Rev Saúde Pública.

[8] Campbell R, Pound P, Morgan M, Daker-White G, Britten N, Pill R (2011). Evaluating meta-ethnography: systematic analysis and synthesis of
qualitative research. Health Technol Assess.

[9] Noblit GW, Hare RD (1988). Meta-ethnography: synthesizing qualitative studies.

[10] Soares CB, Yonekura T (2011). Systematic review of theories: a tool to evaluate and analyze
selected studies. Rev Esc Enferm USP.

[11] Tong A, Sainsbury P, Craig J (2007). Consolidated criteria for reporting qualitative research (COREQ):
a 32-item checklist for interviews and focus groups. Int J Qual Health Care.

[12] Sandelowski M, Barroso J (2007). Handbook for synthesizing qualitative researh.

[13] Sanicola L (2015). As dinâmicas de rede e o trabalho social.

[14] Tong A, Flemming K, McInnes E, Oliver S, Craig J (2012). Enhancing transparency in reporting the synthesis of qualitative
research: ENTREQ.. BMC Med Res Methodol.

[15] Moher D, Liberati A, Tetzlaff J, Altman DG (2009). The PRISMA Group. Preferred reporting items for systematic
reviews and meta-analyses: the PRISMA Statement. PLoS Med.

[16] Ingram J, Johnson D, Hamid N (2003). South Asian grandmothers' influence on breast feeding in
Bistrol. Midwifery.

[17] Masvie H (2006). The role of Tamang mothers-in-law in promoting breast feeding in
Makwanpur District, Nepal. Midwifery.

[18] Kerr RB, Dakishoni L, Shumba L, Msachi R, Chirwa M (2008). We grandmothers know plenty": breastfeeding, complementary
feeding and the multifaceted role of grandmothers in Malawi. Soc Sci Med.

[19] Grassley J, Eschiti V (2008). Grandmother Breastfeeding Support: What Do Mothers Need and
Want?. BIRTH.

[20] Reid J, Schmied V, Beale B (2010). I only give advice if I am asked': examining the grandmother's
potential to influence infant feeding decisions and parenting practices of
new mothers. Women Birth.

[21] Gross FM, Van der Sand ICP, Girardon-Perlini NMO, Cabral FB (2011). Influence of grandmothers on infant feeding: What they say to
their daughters and granddaughters. Acta Paul Enferm.

[22] Silva LR, Cruz LA, Macedo EC, Gomes MN (2013). The influence of grandmothers on breastfeeding of her
grandchildren: beliefs and cultural practices. Rev Pesqui Cuid Fundam.

[23] Premji S, Khowaja S, Meherali S, Forgeron R (2014). Sociocultural influences on newborn health in the first 6 weeks
of life: qualitative study in a fishing village in Karachi,
Pakistan. BMC Pregnancy Childbirth.

[24] Thet MM, Khaing EE, Diamond-Smith N, Sudhinaraset M, Oo S, Aung T (2016). Barriers to exclusive breastfeeding in the Ayeyarwaddy Region in
Myanmar: Qualitative findings from mothers, grandmothers, and
husbands. Appetite.

[25] Innis SM (2014). Impact of maternal diet on human milk composition and
neurological development of infants. Am J Clin Nutr.

[26] Liu H, Hua Y, Luo H, Shen Z, Tao X, Zhu X (2015). An herbal galactagogue mixture increases milk production and
aquaporin protein expression in the mammary glands of lactating
rats. Evidence-Based Complementary Alternative Med.

[27] Srinivas R, Eagappan K, Sasikumar S (2014). The Effect of Naturally Formulated Galactagogue Mix on Breast
Milk Production, Prolactin Level and Short-Term Catch-Up of Birth Weight in
the First Week of Life. IJHSR.

[28] Victora CG, Horta BL, Mola CLM, Quevedo L, Pinheiro RT, Gigante DP (2015). Association between breastfeeding and intelligence, educational
attainment, and income at 30 years of age: a prospective birth cohort study
from Brazil. Lancet.

[29] Frigo J, Zocche DAA, Palavro GL, Turatti LA, Neves ET, Schaefer TM (2015). Perceptions of parents of premature newborns in neonatal
intensive care unit. Rev Enferm UFSM.

[30] Prates LA, Schumalfuss JM, Lipinski JM (2015). Social support network of post-partum mothers in the practice of
breastfeeding. Esc Anna Nery.

[31] Viduedo AFS, Leite JRC, Monteiro JCS, Reis MCG, Gomes-Sponholz FA (2015). Severe lactational mastitis: particularities from
admission. Rev Bras Enferm.

[32] Nduna T (2015). An Explorative Qualitative Study of Experiences and Challenges to
Exclusive Breastfeeding Among Mothers in Rural Zimbabwe. ICAN: Infant Child Adolesc Nutr.

[33] Martins EL, Vargens OMC (2014). Women's perceptions of sexuality while breast-feeding: an
integrative review. Rev Enferm UERJ..

[34] Wambach K, Domian EW, Page-Goertz S, Wurtz H, Hoffman K (2016). Exclusive Breastfeeding Experiences among Mexican American
Women. J Hum Lact.

[35] Gidrewicz DA, Fenton TR (2014). A systematic review and meta-analysis of the nutrient content of
preterm and term breast milk. BMC Pediatrics.

[36] Essa RM, Ismail NIAA (2015). Effect of early maternal/newborn skin-to-skin contact after birth
on the duration of third stage of labor and initiation of
breastfeeding. J Nurs Educ Practice.

[3] Campos AMS, Chaoul CO, Carmona EV, Higa R, Vale IN (2015). Exclusive breastfeeding practices reported by mothers and the
introduction of additional liquids. Rev. Latino-Am. Enfermagem.

[38] Joshi A, Trout KE, Aguirre T, Wilhelm S (2014). Exploration of factors influencing initiation and continuation of
breastfeeding among Hispanic women living in rural settings: a multi-methods
study. Rural Remote Health.

[39] Bernardi MC, Carraro TE, Sebold LF (2011). Puerperal home visit as a strategy for nursing care in primary
health care: integrative review. Rev Rene.

[40] Kornides M, Kitsantas P (2013). Evaluation of breastfeeding promotion, support, and knowledge of
benefits on breastfeeding outcomes. J Child Health Care.

[41] Odom EC, Li R, Scanlon KS, Cria G. Perrine, Grummer-Strawn L (2014). Association of Family and Health Care Provider Opinion on Infant
Feeding with Mother's Breastfeeding Decision. J Acad Nutr Diet.

